# Hypertension in Sub-Saharan Africa: Cross-Sectional Surveys in Four Rural and Urban Communities

**DOI:** 10.1371/journal.pone.0032638

**Published:** 2012-03-12

**Authors:** Marleen E. Hendriks, Ferdinand W. N. M. Wit, Marijke T. L. Roos, Lizzy M. Brewster, Tanimola M. Akande, Ingrid H. de Beer, Sayoki G. Mfinanga, Amos M. Kahwa, Peter Gatongi, Gert Van Rooy, Wendy Janssens, Judith Lammers, Berber Kramer, Igna Bonfrer, Esegiel Gaeb, Jacques van der Gaag, Tobias F. Rinke de Wit, Joep M. A. Lange, Constance Schultsz

**Affiliations:** 1 Department of Global Health, Academic Medical Center, University of Amsterdam, Amsterdam Institute for Global Health and Development, Amsterdam, The Netherlands; 2 Pharmaccess Foundation, Amsterdam, Amsterdam Institute for Global Health and Development, Amsterdam, The Netherlands; 3 Departments of Internal and Vascular Medicine, Academic Medical Center, University of Amsterdam, Amsterdam Institute for Global Health and Development, Amsterdam, The Netherlands; 4 Department of Epidemiology and Community Health, University of Ilorin Teaching Hospital, Ilorin, Nigeria; 5 Pharmaccess Foundation, Windhoek, Namibia; 6 NIMR, Muhimbili Medical Research Centre, Dar es Salaam, Tanzania; 7 MOI University School of Public Health, Eldoret, Kenya; 8 Multidisciplinary Research Centre University of Namibia, Windhoek, Namibia; 9 Amsterdam Institute for International Development, Amsterdam, Amsterdam Institute for Global Health and Development, Amsterdam, The Netherlands; 10 Faculty of Economics and Business Administration, VU University Amsterdam, Amsterdam Institute for Global Health and Development, Amsterdam, The Netherlands; 11 Faculty of Economics and Business Administration, University of Amsterdam, Amsterdam Institute for Global Health and Development, Amsterdam, The Netherlands; 12 Institute of Health Policy and Management, Erasmus University, Rotterdam, The Netherlands; 13 Namibia Institute of Pathology (NIP) Ltd, Windhoek, Namibia; University of Buea, Cameroon

## Abstract

**Background:**

Cardiovascular disease (CVD) is the leading cause of adult mortality in low-income countries but data on the prevalence of cardiovascular risk factors such as hypertension are scarce, especially in sub-Saharan Africa (SSA). This study aims to assess the prevalence of hypertension and determinants of blood pressure in four SSA populations in rural Nigeria and Kenya, and urban Namibia and Tanzania.

**Methods and Findings:**

We performed four cross-sectional household surveys in Kwara State, Nigeria; Nandi district, Kenya; Dar es Salaam, Tanzania and Greater Windhoek, Namibia, between 2009–2011. Representative population-based samples were drawn in Nigeria and Namibia. The Kenya and Tanzania study populations consisted of specific target groups. Within a final sample size of 5,500 households, 9,857 non-pregnant adults were eligible for analysis on hypertension. Of those, 7,568 respondents ≥18 years were included. The primary outcome measure was the prevalence of hypertension in each of the populations under study.

The age-standardized prevalence of hypertension was 19.3% (95%CI:17.3–21.3) in rural Nigeria, 21.4% (19.8–23.0) in rural Kenya, 23.7% (21.3–26.2) in urban Tanzania, and 38.0% (35.9–40.1) in urban Namibia. In individuals with hypertension, the proportion of grade 2 (≥160/100 mmHg) or grade 3 hypertension (≥180/110 mmHg) ranged from 29.2% (Namibia) to 43.3% (Nigeria). Control of hypertension ranged from 2.6% in Kenya to 17.8% in Namibia. Obesity prevalence (BMI ≥30) ranged from 6.1% (Nigeria) to 17.4% (Tanzania) and together with age and gender, BMI independently predicted blood pressure level in all study populations. Diabetes prevalence ranged from 2.1% (Namibia) to 3.7% (Tanzania).

**Conclusion:**

Hypertension was the most frequently observed risk factor for CVD in both urban and rural communities in SSA and will contribute to the growing burden of CVD in SSA. Low levels of control of hypertension are alarming. Strengthening of health care systems in SSA to contain the emerging epidemic of CVD is urgently needed.

## Introduction

Eighty percent of global cardiovascular disease (CVD) mortality occurs in low- and middle-income countries (LMIC) [1], [2]. According to the World Economic Forum, non-communicable diseases (NCD), such as CVD, are a severe threat to global economic development due to the long-term costs of treatment and the negative effects on productivity [3]. The burden of NCD is expected to increase substantially in LMIC and to represent a greater burden of disease compared to communicable diseases by 2030 [4]. The Unites Nations (UN) High level meeting on NCDs which took place in September 2011, emphasized the urgent need for greater measures to prevent and control NCDs in LMIC [5].

The INTERHEART and INTERSTROKE studies showed that modifiable risk factors such as hypertension, obesity, smoking, dyslipidemia and diabetes, account for the majority of CVD in LMIC [6], [7]. Hypertension has been identified as the leading risk factor for mortality worldwide, and is ranked third as a cause of disability-adjusted life-years [8]. Hypertension in SSA is believed to be a problem of the urban areas due to transition to a more Western lifestyle. Early cross-sectional studies from rural areas in SSA report low mean blood pressures that are stable with age, and a low prevalence of hypertension [9], [10], [11], [12], [13]. Migration studies in LMIC showed that migrants from rural to urban areas showed an increase in blood pressure [14], [15]. Recent studies from rural areas in SSA suggest that the prevalence of hypertension is increasing in rural populations. However, unrelated studies are difficult to compare due to a lack of age- standardized prevalence data [16], [17], [18], [19]. Hypertension prevalence data are crucial for understanding the magnitude of the problem, identifying groups at high risk for CVD, and evaluating the effects of policy and practice interventions [20]. However, hypertension prevalence data for SSA are scarce [21], [22]. This study describes the prevalence of hypertension and the determinants of increasing blood pressure in 4 SSA populations in rural Nigeria and Kenya and in urban Namibia and Tanzania.

## Methods

### Ethical clearance

Ethical clearance for the study reported in this paper was obtained from the Ethical Review Committee of the University of Ilorin Teaching Hospital in Nigeria; the Research Ethics Committee at the Ministry of Health and Social Services in Namibia; the National Institute for Medical Research and the Tanzania Commission for Science and Technology in Tanzania, and the Institutional Research and Ethics Committee from Moi Teaching and Referral hospital/Moi University in Kenya. Written informed consent was asked for participation in the blood tests by signature or fingerprint. A standard operating procedure for verbal informed consent was followed for the questionnaire and anthropometrics according to local standard practice for participation in observational studies. Interviewers recorded the outcome of the consent procedure on behalf of the participant. This procedure was approved by the ethical committees and review boards in all four countries.

### Study design and study populations

For this study we included data from four cross-sectional household surveys. The survey in rural Nigeria included a representative, population-based sample of Afon and Ajasse Ipo district in Kwara State. Data were collected in 2009. The survey in rural Kenya included a sample of a specific target group composed of members of dairy farmer cooperatives and their families in the Nandi district. Data were collected in 2011. The survey in urban Tanzania included members of a microcredit organization and their families in Dar es Salaam. Data were collected in 2010. The Namibian survey included a representative sample of the population of Greater Windhoek. The data from Namibia were collected during a survey in 2009 in which households included in a previous survey in 2006, when blood pressure data were not collected, were revisited. Therefore, only the data from the 2009 survey are included in this analysis.

All surveys were carried out as part of an evaluation of the impact of affordable private health insurance schemes [23], [24] on socioeconomic and medical parameters. The surveys in Nigeria, Kenya and Tanzania served as baseline surveys before the insurance scheme was implemented. The Namibian study population had access to a comparatively well-developed market for health insurance [25]. All study populations consisted of private households eligible for the insurance program and similar control populations, excluding household members who at the moment of the visit resided in hospitals, hostels and prisons. Only the adult, non-pregnant population was included in the analysis because hypertension is rare in children and because pregnancy-related hypertension has a different etiology.

### Sampling

**Nigeria:** Stratified random samples were drawn by randomization of geographical areas. A two-staged probability sample was drawn. The first stage consisted of randomly selecting 100 out of 300 enumeration areas (EAs) from the 2005 National Population Census within 15 kilometres distance from three towns: Afon, Aboto Oja - the two towns where the insurance program clinics are based - and Ajasse Ipo. Henceforth, Afon and Aboto Oja are considered treatment area, and Ajasse Ipo control area. In both areas, the sample was stratified based on whether the EA was in or outside this town to allow for sufficient households from remote areas with more difficult access to care. In the “treatment area”, the insurance program will be offered, in the control area, the insurance program will not be rolled out within the next three years. In the second stage, households were sampled weighted on EA size such that the sample represented the population density.

**Kenya:** A random sample of households was drawn from the member list of the dairy farming cooperation that was eligible for the insurance program. The same procedure was followed for the control group, another dairy farming cooperation with similar socio-economic and geographic characteristics who will not be eligible for the insurance program in the coming years.

**Tanzania:** A sample was drawn from the microcredit branch that was eligible for the insurance program using a member list. Out of 159 Market Enterprise Committees (MECs) with a total of 2613 clients, 55 MECs were randomly selected, plus an additional 23 MECs to accommodate possible non-response and refusals. In each MEC, 5 Enterprise groups (EGs) were randomly selected and all group members were included in the treatment sample. A similar branch that was not eligible for the insurance program was used as a control group. A propensity score weighted sample of 55 MECs was drawn from the control branch, in which MECs with similar characteristics as those in the “treatment” branch were more likely to be selected. Weights were determined based on a logit estimation of being in the treatment group. Again 5 EGs were randomly sampled from the selected MECs, and all group members were included in the sample. In total, this resulted in a sample of 1790 clients.

**Namibia:** The survey was conducted among a representative, self-weighted sample of the Greater Windhoek population of Namibia in 2006 using the national sampling frame from the Central Bureau of Statistics in Namibia. In the first stage, 100 Primary Sampling Units (PSUs) were selected proportional to population size. PSU selection was done in proportion to the number of households per cluster from the Census 2001. In the second stage, 20 households per PSU were selected, based on the lists from a household identification exercise. A more detailed description of the survey design and sampling methods is described elsewhere [26]. In 2009, the same households were traced and revisited.

### Sample Size

The sample size for all surveys was determined based on socioeconomic outcome measures defined to study the impact of the insurance program on socioeconomic status. Outcome measures included health care utilization and medical expenses, household assets and household consumption. The main objective of this sub-study, to describe the prevalence of hypertension, was a secondary objective of the four household surveys. Therefore, no formal sample size calculations were performed using the prevalence of hypertension as main outcome measure. However, the sample size in all surveys provides sufficient power to allow for reliable estimations of hypertension prevalence. For example, power to detect a difference in hypertension prevalence across the age groups used in this analysis was 99% in all surveys.

The target sample size was 1500 households in Nigeria, 1200 households in Kenya, 800 households in Tanzania and 2000 households in Namibia.

### Data collection

The methodology of all surveys was based on the World Bank Living Standards Measurements Surveys methodology [27]. Face-to-face questionnaires were administered by trained interviewers recruited from the local universities.

The questionnaire collected demographic, socioeconomic and medical information. Annual household consumption was measured to investigate household socioeconomic status and welfare. Food consumption was measured over the past 7 days, frequent non-food consumption over the past month, and non-frequent non-food consumption over the past year. The consumption score was constructed by summing food consumption (52×), frequent non-food consumption (12×), and non-frequent non-food consumption in each household [27]. Annual per capita consumption was calculated as annual household consumption divided by the number of household members. Local currencies were converted to purchasing power parity (PPP) corrected USD to facilitate comparison across countries using the 2009 World Bank conversion factors [28].

Body weight, height, waist circumference and blood pressure measurements, and a finger prick (venepuncture in Kenya) for blood testing (glucose, cholesterol, HIV, hemoglobin [Hb]) were performed using standardized methods. Blood pressure was measured three times on the upper left arm after at least 5 minutes of rest using a validated automatic blood pressure device (Nigeria, Kenya, Tanzania: OMRON M6 Comfort, OMRON Corporation; Namibia: Rossmax MG 150F, Taiwan). The mean value of the 2^nd^ and 3^rd^ measurement was used for analysis. Weight was measured using validated digital scales (OMRON BF400, OMRON Cooperation; SECA 813, SECA 803, SECA, Germany). Height was measured using SECA portable stadiometers (SECA Germany).

Respondents were asked about their fasting state. Glucose and cholesterol were measured using the following tests. Nigeria and Tanzania: Glucose Whole Blood-OneTouch UltraEasy Monitoring System, Lifescan (Johnson & Johnson company, USA); Namibia: Roche Accutrend GCT and Accutrend Plus Glucose and Cholesterol (Roche Germany); Kenya: Cobas integra 400 plus Chemistry analyzer (Roche Germany). Hemoglobine in Nigeria and Tanzania was measured using Hemocue Whole Blood Hemoglobin Systems (Hemocue, Sweden) and Beckman Coulter AcT 5 Diff Hematology Analyzer (Beckman Coulter Inc, USA) in Kenya. HIV screening was done using the following tests. Nigeria/Kenya: Determine HIV-1/2, (Abbott Japan CO., LTD); Tanzania: SD Bioline HIV 1/2 (SD Standard Diagnostics Inc); Nambia: OraSure® 107 HIV-1 Oral Specimen Collection Device (OraSure Technologies, Inc., Bethlehem, PA), HIV tests: Oral Fluid Vironostika HIV Uni-Form II Micro-ELISA (bioMérieux111 Inc., Durham, NC).

**Revisits:** Households were revisited at least once in all surveys in case household members were not present or in case the interview was not completed during the first visit. In Namibia, households that had relocated their residence between 2006 and 2009 were traced within Greater Windhoek wherever possible. In Kenya, only households in which all household members were absent were revisited.

### Statistical methods

**Definitions:** Hypertension was defined as a measured blood pressure ≥140 mmHg systolic and/or ≥90 mmHg diastolic, or self-reported use of drug treatment for hypertension irrespective of measured blood pressure [29].

Isolated hypertension was defined as a blood pressure of ≥140 mmHg systolic and <90 mmHg diastolic. Isolated diastolic hypertension was defined as a diastolic blood pressure ≥90 mmHg and systolic blood pressure <140 mmHg.

Awareness of hypertension was defined as respondents who self report to have hypertension.

Treatment for hypertension was defined respondents who self report to have hypertension, and who indicate to take drug treatment for hypertension.

Controlled hypertension was defined as respondents who self report to have hypertension, and who have a blood pressure below 140 mmHg systolic and 90 mmHg diastolic.

For 32 (1.8%) respondents who reported hypertension, treatment status was unknown because they had more than 1 chronic disease (for example diabetes and hypertension) and treatment status was only asked for one condition. We considered these respondents as on treatment since this is most likely if they report the diagnosis and receive treatment for another chronic disease.

Diabetes mellitus was defined as the presence of a non-fasting blood glucose of ≥11.1 mmol/L, or a fasting blood glucose of ≥7.0 mmol/L, or self reported use of drug treatment for diabetes [30].

Cholesterol was classified based on the Adult Treatment Panel III classification [31] with high cholesterol defined as ≥6.2 mmol/L.

**Data analysis:** Data were analyzed using STATA, version 10.0 (StataCorp LP, Texas, USA). The prevalence of hypertension and other CVD risk factors was explored using descriptive statistics. Age-standardized prevalence of hypertension was calculated using the WHO New World Reference Population [32]. Age-standardization is necessary when comparing populations given possible differences in the underlying age structure between populations, as well as the strong associating of age with the risk of disease [33]. Although no formal statistical comparison of the hypertension prevalence across the four surveys were performed, age-standardized prevalence rates are provided to allow for comparison with other studies [16].

Ten year CVD risk based on age, gender, blood pressure, diabetes and smoking status was calculated using WHO risk charts for patients ≥40 years of age [34].

Multivariable linear regression analyses were performed to assess the association of well-known predictors for hypertension with blood pressure. Variables included in the analysis were sex, age, body mass index (BMI) and socioeconomic status (measured as consumption, i.e. household foods and non-food expenditures), regardless of statistical significance. In addition, HIV status and Hb were tested because of possible relevance in this setting [35], [36], [37] and were included in the model where significant at a p-value of 0.05. Plausible interactions were explored to assess whether specific combinations of variables modified the risk for increasing blood pressure. In Namibia and Nigeria, estimates were corrected for clustering at the primary sample unit level.

In a second step, the regression models for each population were expanded with additional variables to evaluate if there was clustering of high blood pressure with other risk factors for CVD. Variables included were waist circumference, presence of diabetes, smoking, alcohol use, family history of CVD, blood cholesterol levels and low fruit and vegetable intake. In addition, the association of possible context-specific relevant socioeconomic variables with blood pressure was explored. Variables tested include education, religion and insurance status. Variables included in the final model were significant at a p-value of 0.05.

Respondents without any blood pressure measurement were excluded from the main analysis. A sensitivity analysis was performed to estimate the bias due to non-random missing data. In this analysis, regression models that included age and gender were used to predict the presence of hypertension in respondents without a blood pressure measurement. These imputed values for hypertension presence were used to recalculate the prevalence of hypertension.

## Results

### Population characteristics

**Figure 1:**
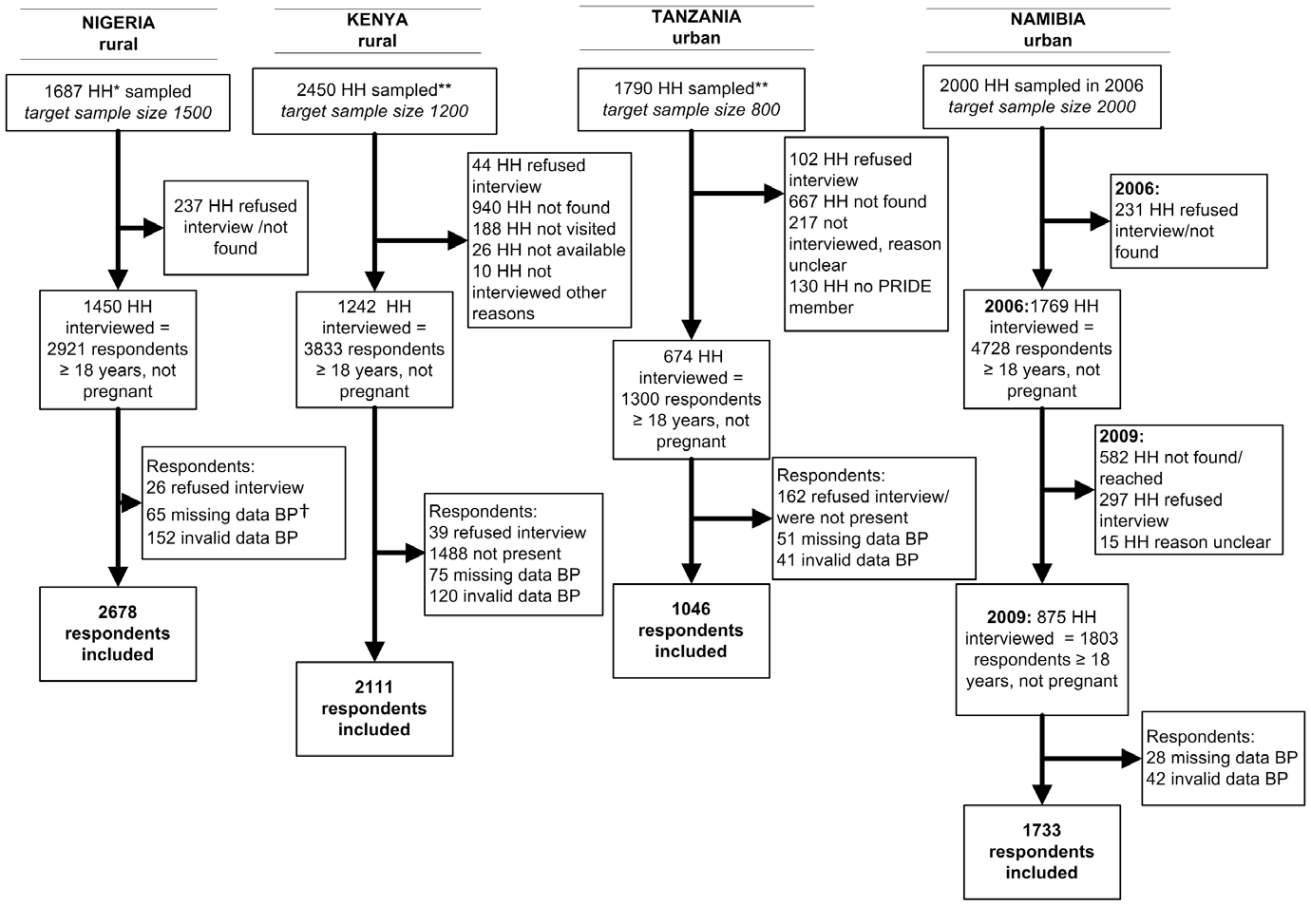
Participation in the 4 surveys (household and individual level). *HH  =  households, **Repeating sampling done because household were difficult to locate due to poor registries used for sampling, ^†^BP =  blood pressure.

The number of sampled households, the number of households and individuals who participated, and reasons for non participation are shown in Figure 1.

The characteristics of the study populations are shown in Table 1. The proportion of males in the sample ranged from 40.1% in Kenya to 46.6% in Nigeria. The mean age distribution of the populations ranged from 36.8 years in Tanzania to 45.3 in Nigeria. The Nigerian respondents mainly belonged to the Yoruba ethnic group (83.2%), the majority of the Kenyan study population consisted of Kalenjin (98.6%). Ethnicity was not asked explicitly in Tanzania and Namibia. However, 83.4% of the Namibian respondents report an indigenous African language as first language which could be used as a proxy to black ethnicity. Only 5 (0.29%) respondents reported German as first language, a proxy for Caucasian descent. The populations were predominantly Muslim in Nigeria (79.4%) and Tanzania (66.3%), and Christian in Kenya (93.4%) and Namibia (98.8%). Education levels were low, especially in the population in Nigeria where >50% of respondents did not complete primary school. Socioeconomic status, measured as per capita annual food and non-food consumption (in PPP corrected USD) was lowest in the Nigerian study population (which was a rural population) and highest in Namibia (an urban population). The variance in consumption level was low in all populations, except for Namibia, as can be expected in rural communities or specific targets groups. Individual insurance coverage of any type of health insurance was low in Nigeria (0.8%) and Tanzania (1.9%). The Namibian sample showed higher coverage levels (26.0%). The Namibian study population had access to a comparatively well-developed market for health insurance [25]. Information on individual health insurance coverage was not available for the Kenyan study population, but 14.8% of the households reported to have at least 1 household member insured.

**Table 1:** Characteristics of the study populations.

	Rural Nigeria		Rural Kenya		Urban Tanzania		Urban Namibia	
	N		N		N		N	
**Male, n (%)**	2678	1247(46.6)	2111	847 (40.1)	1046	423 (40.4)	1733	771 (44.5)
**Age (Years), mean (SD)**	2678	45.3 (18.3)	2109	40.9 (16.2)	1046	36.8 (11.2)	1716	36.9 (13.4)
**Age , n (%)**	2678		2109		1046		1716	
18–24		392 (14.6)		341 (16.2)		143 (13.7)		378 (22.0)
25–34		456 (17.0)		511 (24.2)		328 (31.4)		431 (25.1)
35–44		445 (16.6)		509 (24.1)		340 (32.5)		435 (25.3)
45–54		505 (18.9)		332 (15.7)		164 (15.7)		270 (15.7)
≥55		880 (32.9)		416 (19.7)		71 (6.8)		202 (11.8)
**Level of education, n (%)**	2446		2091		996		1559	
< primary		1457 (59.6)		414 (19.8)		76 (7.6)		302 (19.4)
primary		449 (18.4)		826 (39.5)		685 (68.8)		445 (28.5)
secondary		340 (13.9)		645 (30.8)		202 (20.3)		691 (44.3)
tertiary		200 (8.2)		206 (9.9)		33 (3.3)		121 (7.8)
**Consumption** * **, mean (SD)**	2677	1271.7 (1131.4)	2111	1925.9(2624.8)	1044	3791.6 (2803.0)	1731	4932.0 (9221.5)
**Currently insured, n (%)**	2678	21 (0.8)	NP†		1011	19 (1.9)	1725	448 (26.0)

† NP  =  Not Performed;

* Consumption per capita per year in USD corrected for purchasing power parity (PPP), 2009 conversion rate World bank (http://www.worldbank.org/).

### Prevalence of hypertension and other CVD risk factors

**Figure 2:**
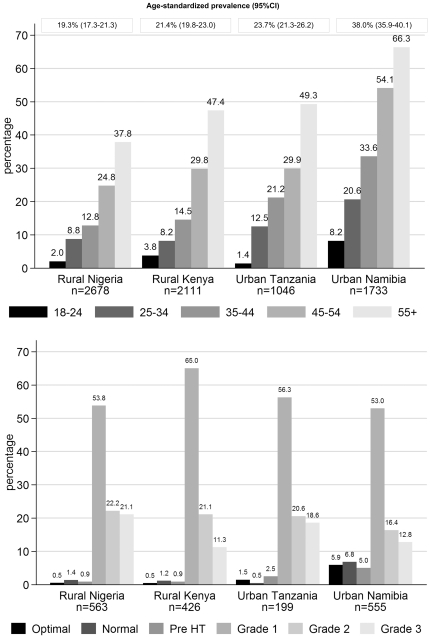
Hypertension prevalence and distribution of blood pressure. 2A: Age-standardized and age stratified hypertension prevalence. 2B: Distribution of blood pressure in patients with hypertension (treated and untreated cases). Optimal  =  systolic blood pressure (SBP) <120 and diastolic blood pressure (DBP) <80; Normal  =  SBP 120–129 and/or DBP 80–84; Pre-HT (hypertension)  =  SBP 130–139 and/or DBP 85–89; Grade 1 =  SBP 140–159 and/or DBP 90–99; Grade 2 =  SBP 160–179 and/or DBP 100–109; Grade 3 =  SBP> = 180 and/or DBP> = 110.

Hypertension was the most prevalent risk factor for CVD in all four populations. The crude prevalence of hypertension ranged from 19.0% in Tanzania to 32.0% in Namibia (Table 2). The age-adjusted prevalence was 19.3% in Nigeria, 21.4% in Kenya, 23.7% in Tanzania and 38.0% in Namibia (Figure 2A). The prevalence of hypertension increased with age (Figure 2A). Within the hypertensive population, the proportion of individuals with grade 2 (≥160/100 mmHg) or grade 3 hypertension (≥180/110 mmHg) (Figure 2B) ranged from 29.2% in Namibia to 43.3% in Nigeria. Mean systolic blood pressure ranged from 121.6 to 123.8 mmHg, mean diastolic blood pressure ranged from 78.1 to 80.2 mmHg. Mean systolic blood pressure in those with hypertension ranged from 142.8 mmHg in Namibia, where the highest treatment rates were found, to 155.6 mmHg in Nigeria (lowest treatment rate). Mean diastolic blood pressure in those with hypertension ranged from 94.3 mmHg in Namibia to 98.2 mmHg in Tanzania. The majority of the hypertensive population had both systolic and diastolic hypertension in all study populations (Table 2). Hypertensive respondents younger than 45 years had predominately diastolic hypertension, whereas the majority of the respondents older than 45 years had both systolic and diastolic hypertension. The proportion of respondents with isolated systolic hypertension increased with age (Figure S1).

**Table 2:** Prevalence of Hypertension and other CVD risk factors.

	Rural Nigeria		Rural Kenya		Urban Tanzania		Urban Namibia	
	N		N		N		N	
**HT** * **(crude), n (%)**	2678	563 (21.0)	2111	426 (20.2)	1046	199 (19.0)	1733	555 (32.0)
Isolated systolic HT†, n (%)	547	113 (20.7)	415	60 (14.5)	190	24 (12.6)	456	59 (12.9)
Isolated diastolic HT†, n (%)	547	102 (18.7)	415	164 (39.5)	190	70 (36.8)	456	155 (14.0)
**WHO CVD risk ≥20% (age ≥40)** ¶ **, n (%)**	1603	79 (4.9)	984	24 (2.4)	380	7 (1.8)	688	17 (2.5)
**Eligible for HT drug treatment WHO** ¶¶ **, n (%)**	2667	244 (9.2)	2090	138 (6.6)	1032	78 (7.6)	1715	162 (9.5)
**SBP** ** **(mmHg) , mean (SD)**	2678	122.3(22.5)	2111	121.6 (18.3)	1046	123.3(17.6)	1733	123.8 (20.2)
**DBP** ** **(mmHg), mean (SD)**	2678	78.1 (13.0)	2111	79.9 (11.7)	1046	79.6 (12.4)	1733	80.2 (14.6)
SBP in HT patients (mmHg) , mean(SD)	563	155.6 (22.3)	426	146.4 (20.8)	199	148.1 (19.4)	555	142.8 (21.0)
DBP in HT patients(mmHg), mean (SD)	563	96.8 (11.7)	426	96.4 (10.6)	199	98.2 (11.4)	555	94.3 (13.5)
**Heart rate (bpm) in those with HT, mean (SD)**	563	78.3 (13.2)	424	74.4 (12.0)	199	83.0 (13.7)		NP∥|
**BMI** †† **(kg/m3), mean (SD)**	2618	22.4 (4.3)	2059	23.2 (4.7)	990	25.8 (4.9)	1710	24.9 (5.4)
**BMI classification, n (%)**	2618		2059		990		1710	
Overweight (>25)		413 (15.8)		433 (21.0)		344 (34.7)		444 (26.0)
Obese (>30)		160 (6.1)		195 (9.5)		172 (17.4)		290 (17.0)
**WC M** ‡ **(cm), mean (SD)**	1232	78.9 (10.4)	796	78.8 (13.0)	420	83.6 (12.3)	769	79.3 (13.1)
**WC F (cm), mean (SD)**	1392	82.6 (13.4)	1231	81.4 (14.7)	615	85.5 (14.4)	953	82.9 (15.4)
**Abdominal obese** § **, n (%)**	2624	410 (15.6)	2027	399 (19.7)	1035	254 (24.5)	1722	341 (19.8)
**DM** ∥ **, n (%)**	1761	51 (2.9)	1331	34 (2.6)	566	21 (3.7)	1652	34 (2.1)
**Glucose (mmol/L), mean (SD)**	1760	5.0 (2.2)	1316	5.0 (1.4)	559	4.9 (2.1)	1651	5.4 (1.3)
**Cholesterol, mean (SD)**	NP		1336	4.4 (1.1)	NP		1714	4.4 (0.7)
**High Cholesterol** § **, n (%)**	NP		1336	64 (4.8)	NP		1714	47 (2.7)
**HIV positive**	1772	50 (2.8)	1329	42 (3.2)	538	80 (14.9)	1679	280 (16.7)
**Hb (mmol/L), mean (SD)**	1762	8.2 (1.4)	1339	9.0 (1.4)	539	7.8 (1.4)	NP	
**Smoking, n (%)**	2669	277 (10.4)	2094	97 (4.6)	1040	43 (4.1)	1733	234 (13.5)
**Alcohol >2 U/day** §§ **, n (%)**	2670	4 (0.1)	2073	29 (1.4)	1033	5 (0.5)	1729	54 (3.1)
**Reported diagnosis of HT, n (%)**	2674	49 (1.8)	2098	84 (4.0)	1023	38 (3.7)	1733	215 (12.4)
**Family History HT, DM, heart disease** *** **, n (%)**	2678	99 (3.7)	2111	319 (15.1)	1046	203 (19.4)	1733	615 (5.5)

* HT  =  hypertension;

† In those with untreated or inadequately treated hypertension;

¶ WHO CVD risk charts start at age 40 years and older;

¶¶ Those with blood pressure ≥160/100 mmHg or 140/90 and 10 year CVD risk of ≥20%;

** SBP  =  systolic blood pressure, DBP  =  diastolic blood pressure;

∥| NP =  not performed;

†† BMI  =  Body Mass Index;

‡ WC  =  waist circumference, M  =  male, F  =  female;

&par DM  = Diabetes Mellitus (non-fasting blood glucose of ≥11.1 mmol/L, or a fasting blood glucose of ≥7.0 mmol/L, or self reported use of drug treatment for DM);

§ High cholesterol ≥6.2 mmol/L;

§§ U = 1 standard unit of alcohol containing approximately 10 g of ethanol;

*** Reported parent with hypertension, diabetes or heart disease.

Sensitivity analyses were performed to estimate the bias due to non-response and loss to follow up on hypertension prevalence in Kenya and Namibia respectively. A prediction model that included age and gender was used to predict the prevalence of hypertension in case of 100% response and 100% follow up. This analysis showed an estimated prevalence of hypertension of 17.3% (95%CI 16.8–17.8) in Kenya, compared to an observed crude prevalence of 20.2% and an estimated prevalence of 27.8% (27.3–28.3) in Namibia, compared to an observed crude prevalence of 32.0%.

**Figure 3:**
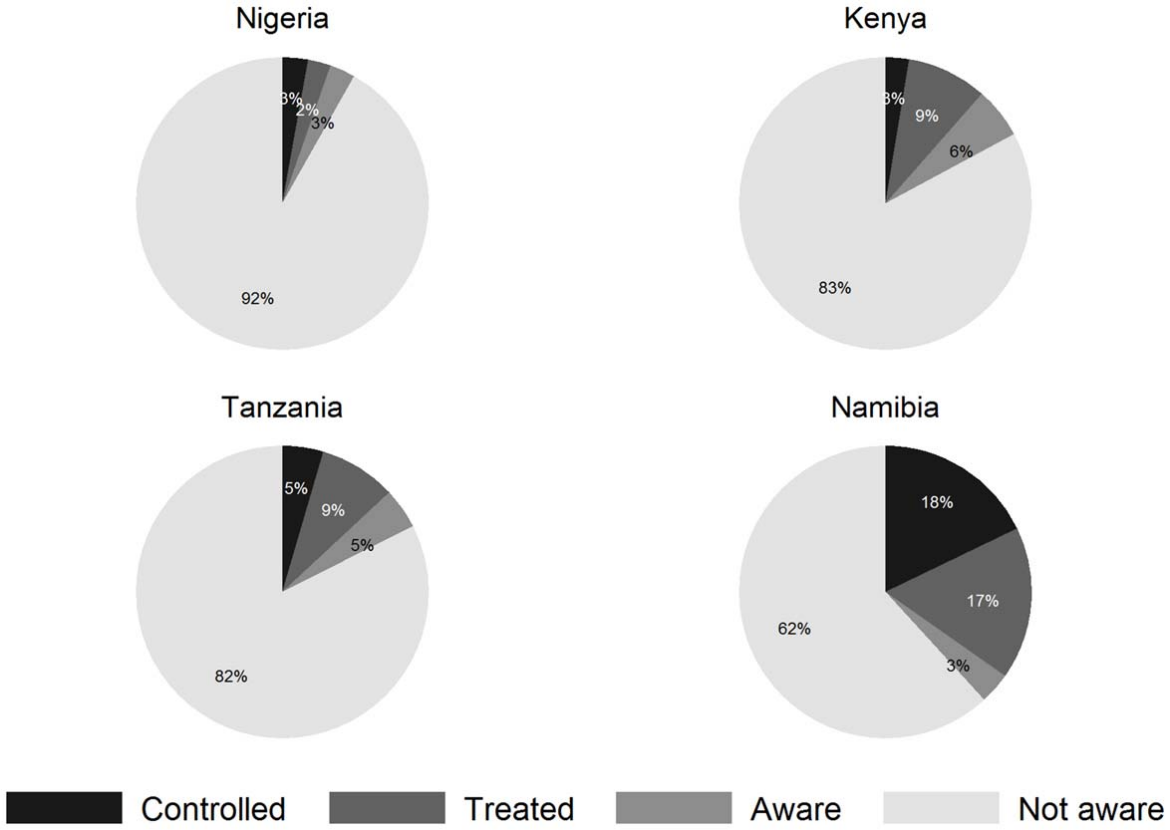
Awareness, treatment and blood pressure control in patients with hypertension. Definitions: Aware  =  respondents who self report to have hypertension, Treated  =  respondents who self report to have hypertension, and who indicate to take drug treatment for hypertension, Controlled  =  respondents who self report to have hypertension, and who have a blood pressure below 140/90 (patients who use drug treatment or for whom treatment status is unknown).

The proportions of respondents with hypertension who were aware of their condition, who reported to be on anti-hypertensive treatment, and whose blood pressure was controlled are shown in Figure 3. The proportions of respondents with hypertension on treatment ranged from 7% in those with grade 1 hypertension to 17.5% in those with grade 3 hypertension. Treatment proportion did not increase with hypertension severity in the Nigerian study population, where the overall treatment proportion was very low (data not shown). Hypertension control was lowest in Kenya (2.6%) and highest in Namibia (17.8%). Respondents who were insured in Namibia showed better hypertension control (Figure S2).

The prevalence of other CVD risk factors was lower compared to the prevalence of hypertension. Obesity prevalence ranged from 6.1% (Nigeria) to 17.4% (Tanzania). Diabetes prevalence was lowest in Namibia (2.1%) and highest in Tanzania (3.7%). Smoking prevalence ranged from 4.1% (Tanzania) to 13.5% (Namibia). Hypercholesterolemia prevalence was 2.7% in Namibia and 4.8% in Kenya (Table 2).

### Risk factors for hypertension

In all populations, age and BMI were independent predictors for increasing blood pressure (Table S1A–D). The effects of increasing age and BMI on blood pressure were different for men and women. Therefore, the effects are reported separately. Women had a steeper increase in blood pressure with increase in age compared to males, while males had a steeper increase in blood pressure with higher BMI. In Namibia, respondents with lower socioeconomic status (lower consumption in USD PPP) had higher blood pressure. Hb concentrations and HIV status were tested because of possible associations with blood pressure and CVD respectively [35], [36], [37]. Hb concentrations were measured in the surveys in Nigeria, Tanzania and Kenya and showed a wide range from 3.6 mmol/L to 13.4 mmol/L. Respondents with Hb in the lowest tertile (by country and gender) had lower blood pressure in Nigeria and Kenya. No association between blood pressure and HIV status was found.

The regression models were expanded to explore associations between increasing blood pressure and other CVD risk factors (Table S1A–D). Respondents with high blood pressure were more likely to have other CVD risk factors such as higher cholesterol and larger waist circumference. Smoking was not associated with blood pressure in any of the study populations but the proportion of smokers was low in general (Table 2). An association between blood glucose levels suspected for diabetes and high blood pressure was only found in the Nigerian population.

In all analyses, adjusting for clustering at household level did not result in significantly different effects (data not shown).

## Discussion

The scale of the surveys described in this study is unique, demonstrating the magnitude of the problem of hypertension in different countries in SSA, including rural and urban areas. The four populations are very different in terms of socioeconomic status, living environment and geographical location. Yet, hypertension was the most frequently observed risk factor for CVD and determinants for blood pressure were similar, in all four populations. The prevalence of hypertension observed in our survey in the Greater Windhoek area in Namibia was remarkably high (crude 32%, age-standardized 38%). This prevalence is similar to the prevalence of hypertension in adults in the USA where an overall prevalence of 31% in adults and 38.6% in non-Hispanic blacks was reported [38]. The prevalence of hypertension in rural Nigeria is lower compared to in high income countries or in other parts of SSA [16], [39], as can be expected in a rural community where people are more likely to follow a traditional African lifestyle. However, both mean blood pressures and the prevalence of hypertension in the Nigerian study population were much higher compared to early studies in rural populations in SSA [40], [41], [42], [43], [44], [45]. The rapid changes towards a more Western lifestyle that are taking place in LMIC is likely to contribute to an increase in the prevalence of hypertension in the coming years, in both rural and urban areas [46]. Whereas mean systolic blood pressure is decreasing since 1980 in high income countries, trends in blood pressure show an increase in systolic blood pressure in many SSA regions and mean systolic blood pressures in SSA are amongst the highest in the world [21]. In addition, people of black African origin have been identified as having a higher risk of target organ damage compared to Caucasians for a given blood pressure [47], [48], [49], [50] and the onset of CVD in LMIC countries occurs at an earlier age compared to high income countries [39]. Finally, even though the prevalence of hypertension in SSA is still lower compared to the high income regions, the large and growing population of LMIC such as Nigeria will result in a considerably larger absolute number of individuals affected compared to high income countries [39], [51]. A recent study in rural Kenya found a similar prevalence of hypertension [52] as our study whilst surveys in urban Tanzania found a higher prevalence [16]. The Tanzanian and Kenyan study populations in our surveys consisted of individuals and their household members, participating in a microcredit program and in a dairy cooperative respectively. These specific characteristics preclude generalization of our conclusions regarding the prevalence of hypertension to the general urban population of Tanzania or the rural population of Kenya. Previous research showed large regional differences in hypertension prevalence in SSA, depending on the level of urbanization and other environmental and possibly genetic factors, although the available data is limited [16], [19], [22]. Therefore, our surveys are not representative for the whole continent of Africa but should be regarded as a contribution to filling the gaps in knowledge on regional prevalence data.

The prevalence of hypertension was based on three measurements of blood pressure on one occasion in this study. In clinical practice, a diagnosis of hypertension requires multiple measurements on several occasions. Therefore, the prevalence of hypertension found in our surveys might be an overestimation, although the normal mean heart rate did not support high blood pressure readings due to sympathic activation. Our study does not allow differentiating between primary and secondary hypertension. To the best of our knowledge, there are no data available on the prevalence of secondary hypertension and the underlying causes in the population from SSA.

Early identification and treatment of people with hypertension is vital. The proportion of respondents with hypertension on treatment was low in Nigeria, Kenya and Tanzania. The proportion on treatment increased with more severe hypertension. Blood pressure control as low as 2.6% (Kenya) is of concern and in line with previous findings from SSA [16], [52], [53], [54], [55], [56], [57], [58]. Poor access to health care, in particular availability and affordability of drugs and travel costs, are barriers to CVD prevention treatment in LMIC. There is an ongoing debate about whether limited funds in LMIC should be spent on NCDs if the burden of communicable diseases is still high [59]. Lifestyle interventions such as salt reduction or weight loss are cheap interventions that might be cost saving [60]. These lifestyle interventions are reported to reduce blood pressure with 3–4 mmHg systolic and 2–3 mmHg diastolic [61], [62]. In our study, between 29.2% (Namibia) and 43.3% (Nigeria) of the respondents with hypertension had grade 2 or grade 3 hypertension. Therefore, our data show that a large proportion of the patients with hypertension may not be adequately treated with lifestyle interventions only. In these groups, drug treatment will be necessary to achieve target goals. Individual (drug) interventions for CVD prevention for high risk groups are available and were shown costs effective in modeling studies [60]. Calculation of total absolute CVD risk according to WHO risk charts did not contribute to identification of respondents eligible for antihypertensive drug treatment other than those with high blood pressure. WHO classifies individuals with blood pressure at or above 140 mmHg systolic or 90 mmHg diastolic eligible for treatment if 10 year CVD risk is ≥20%. Individuals with blood pressure 160 mmHg systolic or 100 mmHg diastolic are eligible for treatment regardless of other risk factors [34]. Due to the low prevalence of diabetes and smoking, treatment eligibility was entirely driven by blood pressure. However, respondents with higher blood pressure were more likely to have other CVD risk factors such as high cholesterol, larger waist circumference and diabetes (Table S1A–D). This clustering of risk factors is important as it increases a persons' overall CVD risk.

Our surveys have all been conducted as part of an evaluation to assess the impact of private health insurance schemes for low and middle income groups in SSA. There is increasing advocacy for affordable health insurance schemes as part of a broader solution to health care financing problems in LMIC [63], [64], [65]. Health insurance may be helpful to improve access to care, especially for patients with chronic conditions such as hypertension. Strengthening of current health systems and improving access to care for patients in LMIC is essential and was recently recommended in the UN declaration resulting from the UN High-Level Meeting on Non-Communicable Diseases [5]. Our data from Namibia showed that treatment and control rates for hypertension were higher for those insured, although no conclusion about improved access to care can be drawn based on these data as people with hypertension might be more likely to enroll in insurance schemes.

We conclude that hypertension is the most frequently observed CVD risk factor in both urban and rural communities in multiple regions in SSA. The prevalence is expected to increase in the coming years. The determinants of blood pressure were consistent in all study populations and similar to described in other populations. Low levels of awareness, treatment and control of hypertension are alarming and may reflect poor access to care. Strengthening of health care systems in SSA to contain the emerging epidemic of CVD is urgently needed.

## Supporting Information

Figure S1 **Blood pressure pattern per age group in respondents with untreated or inadequately treated hypertension, all countries combined.** HT  =  Hypertension, Isolated systolic hypertension  =  systolic blood pressure ≥140 and diastolic blood pressure <90, Isolated diastolic hypertension  =  diastolic blood pressure ≥90 and systolic blood pressure <140.
(TIF)

Figure S2 **Awareness, treatment and blood pressure control in patients with hypertension in Namibia: insured versus not insured.** Definitions: Aware  =  respondents who self report to have hypertension, Treated  =  respondents who self report to have hypertension, and who indicate to take drug treatment for hypertension, Controlled  =  respondents who self report to have hypertension, and who have a blood pressure below 140/90 (patients who use drug treatment or for whom treatment status is unknown).
(TIF)

Table S1 **Multivariable prediction models for blood pressure.** SBP  =  systolic blood pressure, DBP  =  diastolic blood pressure, CI  =  confidence interval, Robust CI in parentheses, NP  =  not performed. Age  =  per year older, BMI  =  per unit increase, waist  =  per cm increase, cholesterol  =  per mmol/L increase *Compared to Hb tertile 1, **Food and non food consumption in log(USD/1000), ^¶^Compared to patients without diabetes, ^§^Compared to no alcohol use, ^∥^per piece increase per week, ^∥∥^Per serving increase per week. Table S1A Step 1: Estimates corrected for ethnic group. Step 2: Estimates corrected for age, gender, BMI, Hb tertile and ethnic group. Table S1B Step 2: Estimates corrected for age, gender, BMI and Hb tertile. Table S1C Step 1 and 2: Estimates corrected for age, gender and BMI Table S1D Step 1: Estimates corrected for ethnic group (language used as proxy). Step 2: Estimates corrected for age, gender, BMI, consumption and ethic group (language used as proxy). Note: Smoking status, family history CVD, education, religion, insurance status, HIV status not significant in any of the study populations (at a p value of 0.05) and excluded from all models. Hemoglobine levels not available for Namibia, cholesterol levels only available for Kenya and Namibia, data on fruit and vegetable intake only available for Tanzania and Kenya.
(DOC)
