# Supplementary figures and images for: Hypertension in Sub-Saharan Africa: Cross-Sectional Surveys in Four Rural and Urban Communities
Source: PLoS One. 2012 Mar 12;7(3):e32638. doi: 10.1371/journal.pone.0032638 (PMC3299675; doi:10.1371/journal.pone.0032638)

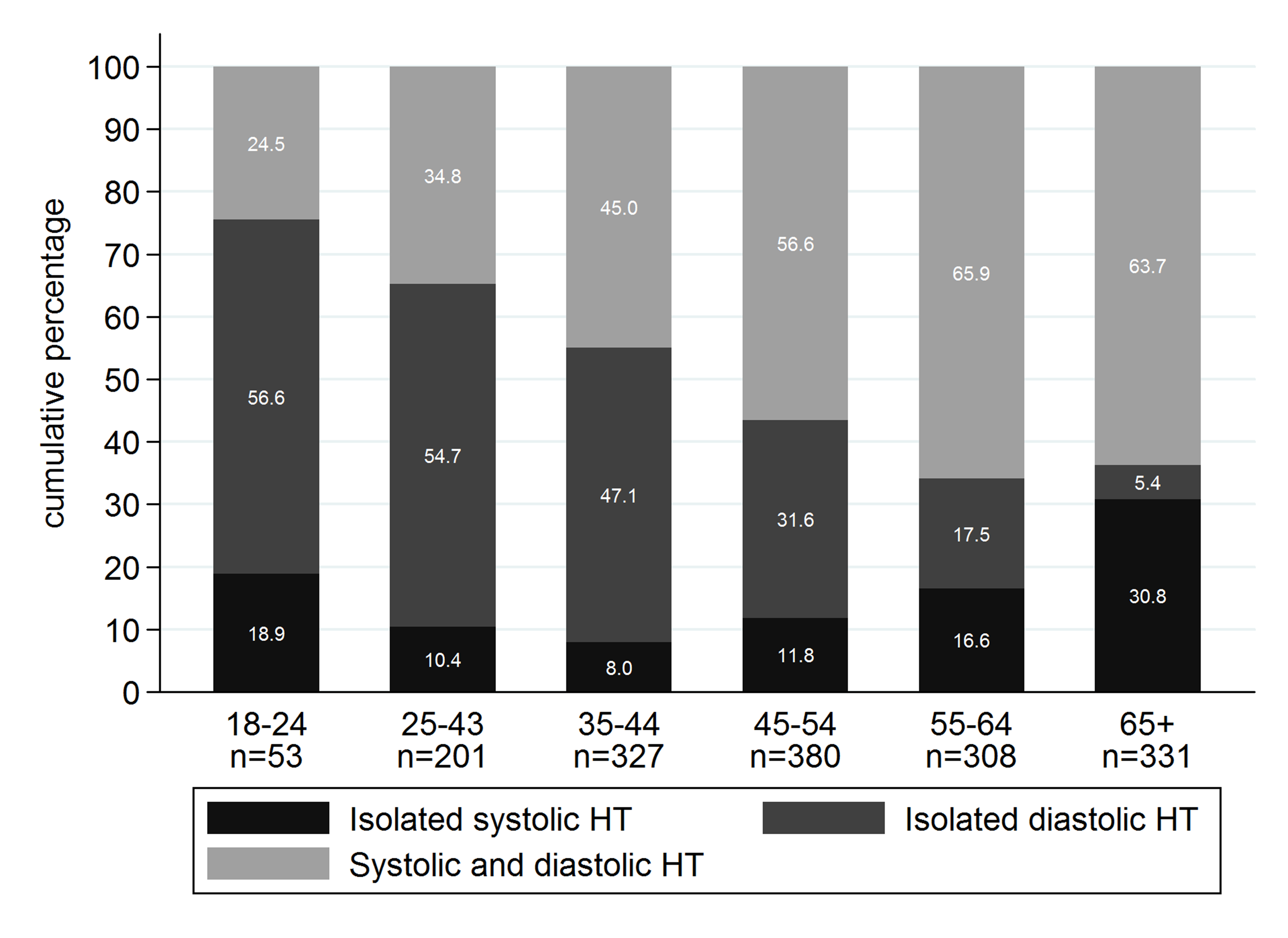

Supplement: Figure S1 — Blood pressure pattern per age group in respondents with untreated or inadequately treated hypertension, all countries combined. HT = Hypertension, Isolated systolic hypertension = systolic blood pressure ≥140 and diastolic blood pressure <90, Isolated diastolic hypertension = diastolic blood pressure ≥90 and systolic blood pressure <140. (TIF) [file pone.0032638.s001.tif]

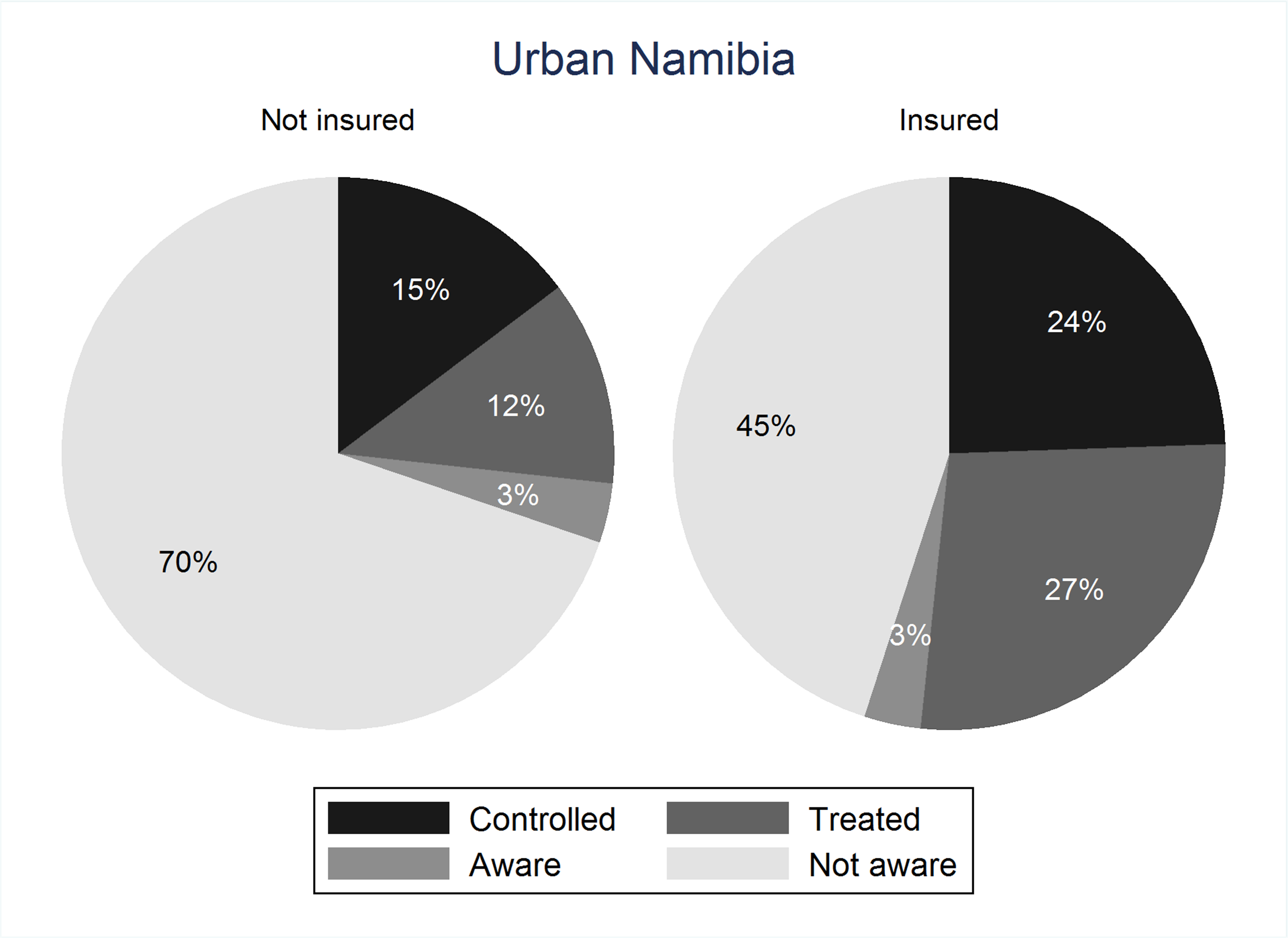

Supplement: Figure S2 — Awareness, treatment and blood pressure control in patients with hypertension in Namibia: insured versus not insured. Definitions: Aware = respondents who self report to have hypertension, Treated = respondents who self report to have hypertension, and who indicate to take drug treatment for hypertension, Controlled = respondents who self report to have hypertension, and who have a blood pressure below 140/90 (patients who use drug treatment or for whom treatment status is unknown). (TIF) [file pone.0032638.s002.tif]
