# Supplementary material for: Hypertension in Sub-Saharan Africa: Cross-Sectional Surveys in Four Rural and Urban Communities
Source: PLoS One. 2012 Mar 12;7(3):e32638. doi: 10.1371/journal.pone.0032638 (PMC3299675; doi:10.1371/journal.pone.0032638)
Supplement: Table S1 — Multivariable prediction models for blood pressure. SBP = systolic blood pressure, DBP = diastolic blood pressure, CI = confidence interval, Robust CI in parentheses, NP = not performed. Age = per year older, BMI = per unit increase, waist = per cm increase, cholesterol = per mmol/L increase *Compared to Hb tertile 1, **Food and non food consumption in log(USD/1000), ¶Compared to patients without diabetes, §Compared to no alcohol use, ∥per piece increase per week, ∥∥Per serving increase per week. Table S1A Step 1: Estimates corrected for ethnic group. Step 2: Estimates corrected for age, gender, BMI, Hb tertile and ethnic group. Table S1B Step 2: Estimates corrected for age, gender, BMI and Hb tertile. Table S1C Step 1 and 2: Estimates corrected for age, gender and BMI Table S1D Step 1: Estimates corrected for ethnic group (language used as proxy). Step 2: Estimates corrected for age, gender, BMI, consumption and ethic group (language used as proxy). Note: Smoking status, family history CVD, education, religion, insurance status, HIV status not significant in any of the study populations (at a p value of 0.05) and excluded from all models. Hemoglobine levels not available for Namibia, cholesterol levels only available for Kenya and Namibia, data on fruit and vegetable intake only available for Tanzania and Kenya. (DOC) [file pone.0032638.s003.doc]

**S1A**

| **Rural Nigeria** | SBP mmHg | DBP mmHg |
| --- | --- | --- |
|  | Regression Coefficients (95% CI) | Regression Coefficients (95% CI) |
| **Step 1: Predictors of blood pressure** |  |  |
| Female: Age | 0.74 (0.67 - 0.81) | 0.31 (0.27 - 0.34) |
| Male: Age | 0.34 (0.28 - 0.40) | 0.18 (0.14 - 0.22) |
| Female: BMI | 0.54 (0.32 - 0.75) | 0.65 (0.51 - 0.79) |
| Male: BMI | 1.40 (1.15 - 1.65) | 0.86 (0.70 - 1.02) |
| Hb tertile 2, mmol/l* | 0.88 (-1.22 - 2.98) | 1.57 (0.21 - 2.93) |
| Hb tertile 3, mmol/l* | 2.04 (-0.12 - 4.20) | 2.12 (0.70 - 3.53) |
| Consumption** | 0.58 (-0.81 - 1.96) | -0.26 (-1.04 - 0.52) |
| Observations | 2617 | 2617 |
| **Step 2: Clustering of CVD risk factors** |  |  |
| Female: Waist, cm | -0.039 (-0.16 - 0.082) | 0.026 (-0.045 - 0.096) |
| Male: Waist, cm | 0.037 (-0.066 - 0.14) | 0.029 (-0.037 - 0.094) |
| Diabetes^¶^ | 10.7 (2.53 - 18.9) | 6.82 (2.91 - 10.7) |
| Observations | 2570 | 2570 |

**S1B**

| **Rural Kenya** | SBP mmHg | DBP mmHg |
| --- | --- | --- |
|  | Regression Coefficients (95% CI) | Regression Coefficients (95% CI) |
| **Step 1: Predictors of blood pressure** |  |  |
| Female: Age | 0.54 (0.46 - 0.61) | 0.22 (0.18 - 0.27) |
| Male: Age | 0.39 (0.31 - 0.48) | 0.22 (0.17 - 0.27) |
| Female: BMI | 0.44 (0.25 - 0.63) | 0.59 (0.47 - 0.71) |
| Male: BMI | 1.00 (0.76 - 1.24) | 0.65 (0.50 - 0.81) |
| Hb tertile 2, mmol/l* | 1.07 (-0.99 - 3.14) | 2.21 (0.87 - 3.56) |
| Hb tertile 3, mmol/l* | 2.52 (0.47 - 4.57) | 3.56 (2.16 - 4.96) |
| Consumption** | 0.032 (-0.90 - 0.96) | 0.21 (-0.40 - 0.82) |
| Observations | 2057 | 2057 |
| **Step 2: Clustering of CVD risk factors** |  |  |
| Female: Waist, cm | -0.047 (-0.13 - 0.033) | 0.070 (0.017 - 0.12) |
| Male: Waist, cm | -0.053 (-0.19 - 0.082) | 0.045 (-0.026 - 0.12) |
| Cholesterol, mmol/L | 1.11 (0.37 - 1.84) | 0.85 (0.32 - 1.39) |
| Alcohol < 1 U p/day^§^ | 1.40 (-2.83 - 5.63) | 2.87 (-0.23 - 5.98) |
| Alcohol 1-2 U p/day^§^ | 3.51 (-2.25 - 9.26) | 6.19 (2.20 - 10.2) |
| Alcohol > 2 U p/day^§^ | 2.91 (-5.93 - 11.7) | 5.17 (0.12 - 10.2) |
| Observations | 1947 | 1947 |

**S1C**

| **Urban Tanzania** | SBP mmHg | DBP mmHg |
| --- | --- | --- |
|  | Regression Coefficients (95% CI) | Regression Coefficients (95% CI) |
| **Step 1: Predictors of blood pressure** |  |  |
| Female: Age | 0.78 (0.63 - 0.93) | 0.44 (0.35 - 0.53) |
| Male: Age | 0.61 (0.44 - 0.77) | 0.39 (0.29 - 0.49) |
| Female: BMI | 0.31 (0.050 - 0.57) | 0.34 (0.17 - 0.51) |
| Male: BMI | 0.76 (0.41 - 1.11) | 0.41 (0.18 - 0.64) |
| Hb tertile 2, mmol/l* | -0.40 (-4.06 - 3.26) | 0.57 (-2.01 - 3.16) |
| Hb tertile 3, mmol/l* | -1.88 (-5.62 - 1.85) | -0.45 (-3.07 - 2.17) |
| Consumption** | 0.55 (-1.10 - 2.19) | 0.82 (-0.40 - 2.04) |
| Observations | 988 | 988 |
| **Step 2: Clustering of CVD risk factors** |  |  |
| Female: Waist, cm | -0.041 (-0.17 - 0.087) | 0.14 (0.047 - 0.24) |
| Male: Waist, cm | 0.41 (0.25 - 0.56) | 0.33 (0.20 - 0.46) |
| Fruit|| | -0.094 (-0.25 - 0.059) | -0.13 (-0.24 - -0.026) |
| Vegetables|||| | -0.14 (-0.30 - 0.017) | -0.13 (-0.25 - -0.016) |
| Observations | 971 | 971 |

**S1D**

| **Urban Namibia** | SBP mmHg | DBP mmHg |
| --- | --- | --- |
|  | Regression Coefficients (95% CI) | Regression Coefficients (95% CI) |
| **Step 1: Predictors of blood pressure** |  |  |
| Female: Age | 0.63 (0.51 - 0.75) | 0.34 (0.26 - 0.42) |
| Male: Age | 0.44 (0.32 - 0.55) | 0.38 (0.30 - 0.47) |
| Female: BMI | 0.57 (0.30 - 0.83) | 0.45 (0.28 - 0.63) |
| Male: BMI | 1.08 (0.80 - 1.36) | 0.51 (0.31 - 0.71) |
| Hb tertile 2, mmol/l* | NP | NP |
| Hb tertile 3, mmol/l* | NP | NP |
| Consumption** | -1.50 (-2.48 - -0.52) | -1.28 (-2.07 - -0.49) |
| Observations | 1691 | 1691 |
| **Step 2: Clustering of CVD risk factors** |  |  |
| Female: Waist, cm | 0.17 (0.048 - 0.30) | 0.12(0.035 - 0.21) |
| Male: Waist, cm | 0.25 (0.11 - 0.40) | 0.048 (-0.075 - 0.17) |
| Cholesterol, mmol/L | 1.28 (-0.16 - 2.73) | 1.30 (0.27 - 2.33) |
| Alcohol < 1 U p/day^§^ | -0.25 (-2.14 - 1.64) | 0.13 (-1.38 - 1.65) |
| Alcohol 1-2 U p/day^§^ | 2.33 (-2.11 - 6.76) | 4.23 (0.46 - 8.00) |
| Alcohol > 2 U p/day^§^ | 6.77 (0.66 - 12.9) | 4.44 (0.23 - 8.65) |
| Observations | 1681 | 1681 |
